# ALK alterations in salivary gland carcinomas

**DOI:** 10.1007/s00428-020-02971-w

**Published:** 2020-11-25

**Authors:** Hanna Majewska, Adam Gorczyński, Piotr Czapiewski, Roopika Menon, Judith Mueller, Sotirios Lakis, Johannes M. Heuckmann, Jan Laco, Ruta Gupta, Simon Andreasen, Dominik Stodulski, Mariola Iliszko, Rafał Dziadziuszko, Jacek Jassem, Lukas C. Heukamp, Wojciech Biernat

**Affiliations:** 1 Department of Pathomorphology, Medical University of Gdańsk, ul. Smoluchowskiego, 17 80-211 Gdańsk, Poland; 2 NEO New Oncology GmbH, Cologne, Germany; 3 The Fingerland Department of Pathology, Charles University Faculty of Medicine in Hradec Kralove and University Hospital Hradec Kralove, Hradec Kralove, Czech Republic; 4 Department of Tissue Pathology and Diagnostic Oncology, University of Sydney, Sydney, Australia; 5 Department of Otorhinolaryngology and Maxillofacial Surgery, Køge University Hospital, Køge, Denmark; 6 Department of Otolaryngology, Medical University of Gdańsk, Gdańsk, Poland; 7 Department of Biology and Medical Genetics, Medical University of Gdańsk, Gdańsk, Poland; 8 Department of Oncology and Radiotherapy, Medical University of Gdańsk, Gdańsk, Poland; 9 Institute of Hematopathology Hamburg, Hamburg, Germany

**Keywords:** Anaplastic lymphoma kinase, Salivary gland carcinoma, Intraductal carcinoma, FISH, Immunohistochemistry, Next generation sequencing

## Abstract

Salivary gland carcinomas represent a heterogeneous group of poorly characterized head and neck tumors. The purpose of this study was to evaluate *ALK* gene and protein aberrations in a large, well-characterized cohort of these tumors. A total of 182 salivary gland carcinomas were tested for anaplastic lymphoma kinase (ALK) positivity by immunohistochemistry (IHC) using the cut-off of 10% positive cells. ALK positive tumors were subjected to FISH analysis and followed by hybrid capture–based next generation sequencing (NGS). Of the 182 tumors, 8 were ALK positive by IHC. Further analysis using hybrid capture NGS analysis revealed a novel *MYO18A* (Exon1-40)-*ALK* (exon 20-29) gene fusion in one case of intraductal carcinoma. Additional genomic analyses resulted in the detection of inactivating mutations in *BRAF* and *TP53*, as well as amplifications of *ERBB2* and *ALK*. *ALK* rearrangements are a rare entity in salivary gland carcinomas. We identified a potentially targetable novel *ALK* fusion in an intraductal carcinoma of minor salivary glands.

**Supplementary Information:**

The online version contains supplementary material available at 10.1007/s00428-020-02971-w.

## Introduction

Salivary gland tumors comprise a heterogeneous group of benign and malignant neoplasms, accounting for approximately 6% of all head and neck cancers. Salivary gland carcinomas (SGCs) are rare and characterized by extensive morphological diversity and variable clinical behavior [1].

Molecular genetic studies have recently revealed that gene rearrangements play a significant role in the molecular pathogenesis of SGCs [1]. These translocations are associated with specific histological subtypes or with particular morphological patterns. For instance, the translocations t(11;19) and t(11;15), resulting in *CRTC1*-*MAML2* or *CRTC3*-*MAML2* fusion oncogenes, respectively, are relatively common in mucoepidermoid carcinomas (MECs) originating in diverse anatomical locations [2–4]. *ETV6-NTRK3* and *ETV6-RET* translocation was found to be specific for secretory carcinoma (a.k.a. mammary analogue secretory carcinoma, MASC) and has not been documented in any other salivary gland tumor [5, 6]. Around 80–90% of adenoid cystic carcinomas (AdCC) reveal *MYB* or *MYBL1* activation by gene fusion, leading to overexpression of *MYB-NFIB* or *MYBL1-NFIB* fusion protein, respectively [3, 7, 8]. Rearrangements of *EWSR1* were found in some tumors with clear cell morphology (e.g., in hyalinizing clear cell carcinoma (HCCC) and clear cell odontogenic carcinoma [CCOC]) and, most recently, in a subset of clear cell myoepithelial carcinoma (CCMC) characterized by more aggressive clinical behavior [9, 10]. These alterations are functionally and clinically important biomarkers and may be considered new targets for personalized therapies.

ALK is a tyrosine kinase receptor that was originally described in 1994 by Morris et al. [11] in anaplastic large cell lymphoma (ALCL). Since then, its aberrations, such as rearrangements and gene copy number gains, have been found in many other malignant and benign neoplasms, e.g., *EML4-ALK* translocations occurring in 2–7% of lung adenocarcinomas [12]. There has been a growing interest in the pathogenic role of ALK alterations in other malignancies, as patients with ALK-positive tumors may benefit from therapies with ALK tyrosine kinase inhibitors. These alterations have been identified, among others, in benign fibrous histiocytoma [13], papillary thyroid carcinoma [14, 15], sarcomatoid carcinoma of the head and neck [16], and pediatric kidney carcinoma [17]. Likewise, there have been described five SGCs with *ALK* alterations until now: a myoepithelial carcinoma with *MSN-ALK* rearrangement [18], a secretory carcinoma with *CTNNA1-ALK* rearrangement [19], an intraductal carcinoma with *STRN-ALK* rearrangement [20], and two salivary gland carcinomas—one with *HNRNPH3-ALK* and another with *EML4-ALK* gene fusion [21]. Therefore, we systematically evaluated ALK by immunohistochemistry (IHC) followed by dual color fluorescence in situ hybridization (FISH) and new generation sequencing (NGS) in IHC-positive cases in a large, well-characterized series of salivary gland carcinomas.

## Materials and methods

The study material comprised the consecutive historical series of primary carcinomas of major and minor salivary glands resected at the Medical University of Gdańsk (Departments of Otolaryngology and Maxillofacial Surgery) between 1992 and 2012. A total of 182 salivary gland carcinomas (Table 1, Supplementary Table S1) was reviewed and reclassified according to the criteria published by WHO in 2017, with application of molecular testing, if necessary [5, 22]. After obtaining preliminary results, seven additional intraductal carcinomas were included into the study as obtained from the collections of some authors (SA, case nos. 1–3; RS, case no. 4; JL, case nos. 5–6; RG, case no. 7).

**Table 1:** Salivary gland carcinomas analyzed in the study

Histopathologic type	Number of cases (%)
Adenoid cystic carcinoma	61 (33.5%)
Mucoepidermoid carcinoma	23 (12.6%)
Carcinoma ex pleomorphic adenoma	24 (13%)
Acinic cell carcinoma	15 (8.2%)
Adenocarcinoma not otherwise specified	10 (5.5%)
Salivary duct carcinoma	10 (5.5%)
Polymorphous adenocarcinoma	7 (3.8%)
Mammary analogue secretory carcinoma	7 (3.8%)
Epithelial-myoepithelial carcinoma	6 (3.3)
Basal cell adenocarcinoma	4 (2.2%)
Undifferentiated carcinoma	3 (1.6%)
Squamous cell carcinoma	3 (1.6%)
Myoepithelial carcinoma	2 (1.1%)
Neuroendocrine carcinoma	2 (1.1%)
Papillary cystadenocarcinoma	2 (1.1%)
Lymphoepithelial carcinoma	1 (0.5%)
Cribriform adenocarcinoma	1 (0.5%)
Intraductal carcinoma	1 (0.5%)

In all cases, paraffin blocks and recuts were available for histological, IHC, and molecular analysis.

### Tissue microarrays

Tissue microarrays (TMAs) were generated from formalin-fixed paraffin-embedded (FFPE) surgical resection tumor specimens (*n* = 182) and control samples (tonsils *n* = 2, liver *n* = 1, ALK positive adenocarcinoma of the lung *n* = 1). Briefly, morphologically representative areas of tumors were targeted on H&E sections of donor blocks. Four 1.0-mm cores were obtained from each tumor and brought into a recipient paraffin block forming microarray blocks using tissue-arraying instrument (MTA-I, Beecher Instruments).

### Immunohistochemical analysis

For IHC, 4-μm-thick TMA sections were cut, mounted on silanized slides, and subjected to heat-induced epitope retrieval by immersion in EnVision Flex Target Retrieval Solution, High pH, at pH 9, at 97 °C in the PT Link (Dako) for 20 min. The staining was performed by Autostainer Link 48, DAKO, with the use of primary mouse monoclonal anti-ALK antibody (clone 5A4, NCL-ALK, Novocastra) according to the manufacturer’s instructions. Briefly, endogenous peroxidase was blocked by a 5-min treatment with EnVision Flex Peroxidase-Blocking Reagent. To visualize the reaction, the EnVision Flex/HRP (30 min) and DAB substrate working solution (10 min) were used. Finally, the slides were counterstained with hematoxylin. An appropriate positive control (one case of ALK-positive adenocarcinoma of the lung) was included in the staining procedure.

The IHC staining of ALK was expressed as the percentage of cells with positive staining, considering different cellular compartments (membranous, cytoplasmic, and nuclear). The intensity of staining was graded as follows: 0 for absent, 1 weak, 2 moderate, and 3 strong. Staining was considered positive if it was moderate to strong and detected in more than 10% of neoplastic cells in any compartment.

### FISH

Eight SGC samples being ALK-positive by IHC in more than 10% of cells were subjected to dual color break apart FISH analysis. The 4-μm-thick FFPE sections were cut and mounted on silanized slides. The 4-μm-thick FFPE sections were cut and mounted on silanized slides. Hybridization was carried out according to the protocol provided by the manufacturer, using the VYSIS LSI ALK Dual Color Break Apart FISH Probe (Abbott Molecular, Abbott Park, IL, USA). The slides were deparaffinized in xylene, dehydrated by gradient alcohol, and rehydrated in deionized water, heated in the × 1 target retrieval solution (pH 6) (DAKO, Glostrup, Denmark) for 120 min at 60 °C and subsequently cooled for 20 min at room temperature in the same solution. The slides were washed in deionized water for 5 min and immersed in the protease solution (20 mg/ml) for 20 min at 37 °C. Subsequently, they were placed into deionized water for 3 min, dehydrated in a series of ethanol solution (70%, 85%, and 96% for 2 min each), and air-dried. An appropriate amount of FISH probe was applied onto each specimen, which was then covered with a glass cover slip and sealed with rubber cement. The slides were incubated in the HYBrite^TM^ instrument (Vysis) with co-denaturation parameters at 73 °C for 3 min and hybridization parameters at 37 °C for 14 h. The rubber-cemented cover slips were then removed, and the slides were placed in a post-hybridization wash solution (2xSSC/0.3% NP-40) at 74 °C for 2 min. The slides were air-dried in the dark, counterstained with DAPI II (VYSIS/Abbott), cover slipped, and immediately examined.

Hybridized slides were examined with an Imaginer Z2 (Zeiss, Germany) fluorescence microscope using a × 100 objective and filter sets, triple band pass (DAPI/Spectrum Green/Spectrum Orange), dual band pass (FITC/Texas Red), and single band pass (Spectrum Green or Spectrum Orange). One hundred randomly selected non-overlapping tumor cell nuclei were examined for the presence of green and red fluorescent signals. Samples were classified as positive for ALK rearrangement if 15% or more of nuclei showed split signals (e.g., red and green signals were separated by ≥ 2 signal diameters) or single red signals (3′ALK).

### Next generation sequencing

Genomic DNA was extracted from the salivary gland FFPE block, sheared (Covaris), and subjected to hybrid capture–based NGS panel of 79 genes (Supplementary Table S2) to detect point mutations, small insertions and deletions, copy number alterations, and genomic rearrangements in a single assay (NEO New Oncology GmbH, Cologne, Germany). Briefly, after shearing, adapters were ligated, and individual genomic regions of interest were enriched using complementary bait sequences (hybrid capture procedure). The selected baits ensure optimal coverage of all relevant genomic regions. After enrichment, all targeted fragments were amplified (clonal amplification) and sequenced in parallel at high sequencing depth. Computational analysis was performed using NEO New Oncology’s proprietary computational biology analysis to detect relevant genomic alterations in a quantitative manner.

## Results

### Clinical and histopathologic data of ALK FISH–positive tumor

The only *ALK*-rearranged tumor occurred in the minor salivary gland of the upper lip of a 73-year-old female. Her previous medical history was non-contributory. The tumor was palpable for about 10 years and was painful under pressure. The patient underwent radical tumor resection with clear surgical margins and is alive with no evidence of disease after 8 years of follow-up.

**Fig. 1:**
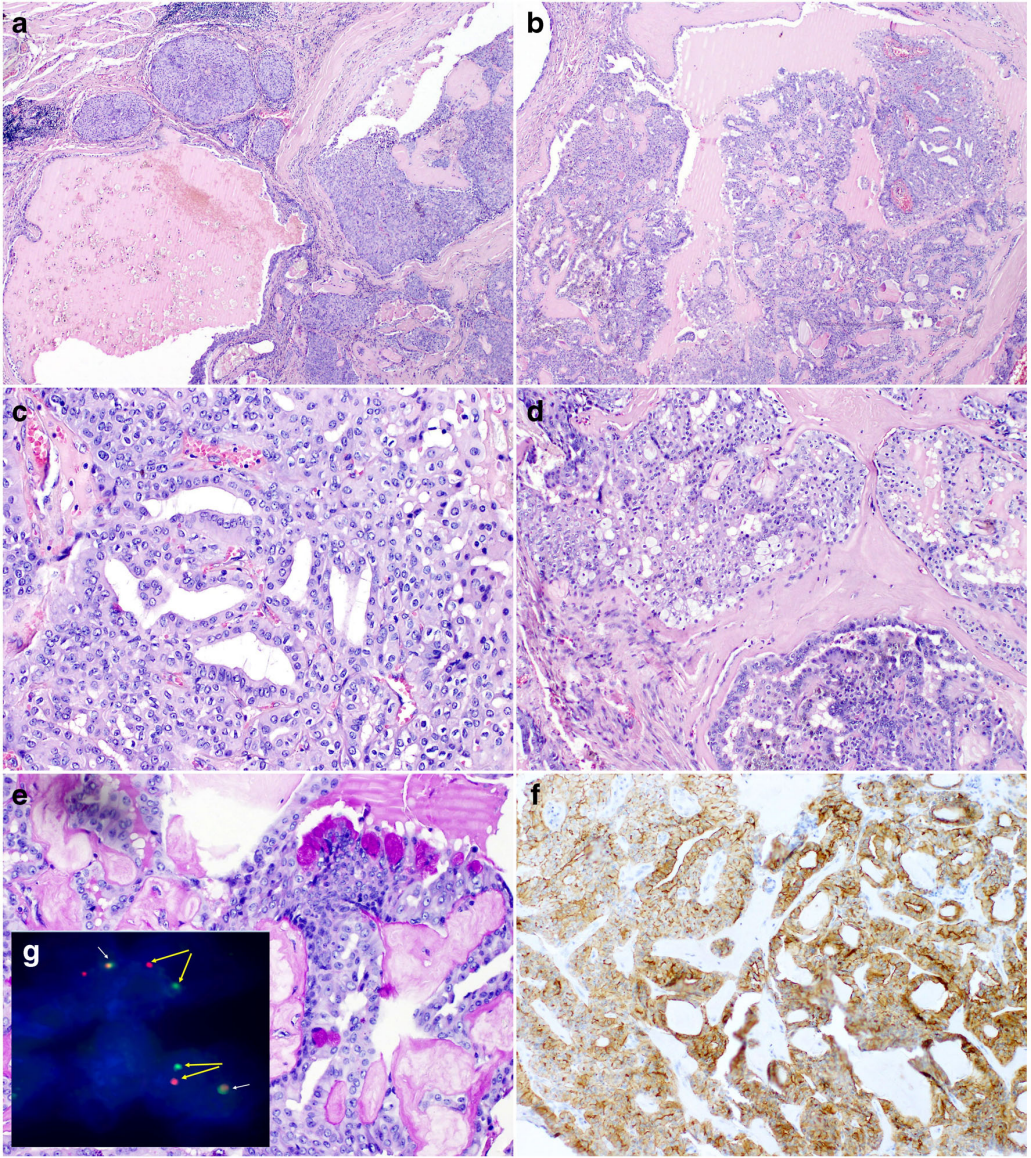
Intraductal carcinoma. **a** The unencapsulated tumor composed of variably sized cysts and nests with mainly intraductal proliferations The neoplastic nests contain epithelioid cells with abundant eosinophilic cytoplasm and regular oval and round nuclei with conspicuous nucleoli (H&E × 20). **b** The cysts contained micropapillary structures, solid areas with irregular fenestrations or cribriform areas(H&E × 20). **c–d** The neoplastic nests contain epithelioid cells with abundant eosinophilic cytoplasm and regular oval and round nuclei with conspicuous nucleoli (**c** H&E × 200; **d** H&E × 100). **e** Focal mucinous differentiation with PAS-positive vacuoles in the cytoplasm (H&E × 200). **f** Immunohistochemical staining showed strong membranous expression of ALK in 100% of cells (H&E × 200). **g** Fluorescence in situ hybridization with ALK Dual Color Break Apart FISH Probe. Nuclei with split red and green signals indicate ALK break (yellow arrows). Chromosome 2 with normal gene shows yellow signal (overlapping green and red and white arrows)

Histologically, this case featured a histomorphology of intraductal carcinoma (IC), intercalated duct type, and was characterized by predominantly macrocystic and microcystic with focally solid growth pattern; it had a multilobular architecture divided by thin or hyalinized fibrous septa (Fig. 1a–d). Intraluminal PAS-positive secretions were visible in some portions of the tumor (Fig. 1e). Tumor cells were diffusely positive for CK7, S100 protein, SOX10, and mammaglobin. The tumor islands were consistently surrounded by a layer of myoepithelial cells as determined by p63, CK14, and calponin expression. On high-power magnification, the tumor revealed bland cytological features, with tumor cells ranging from small to medium size. They had indistinct cell borders and round or ovoid nuclei with dark condensed or finely dispersed chromatin and large pale to eosinophilic cytoplasm. Isolated mucinous cell were scattered in the neoplasm (Fig. 1e). Proliferative activity was low, with a Ki67/MIB1 index < 5%.

### Pathology, immunohistochemistry, and FISH

Study material included 18 pathologic types of salivary gland tumors (Table 1). The IHC staining of the ALK protein was considered positive in eight cases (Table 2). The positive cases were subjected to FISH analysis and included the following tumor types: AdCC (three cases), basal cell carcinoma (BCAC, two cases), and one case each of myoepithelial carcinoma (MYEC), polymorphous adenocarcinoma (PAC), cribriform adenocarcinoma of minor salivary glands (CAMSG), intraductal carcinoma, and acinic cell carcinoma (AciCC). One case (intraductal carcinoma) displayed strong membranous expression of ALK in 100% of cells (Fig. 1f) and was subsequently shown to be positive for *ALK* rearrangement by FISH analysis (Fig. 1g). The remaining seven cases were FISH negative.

**Table 2:** Characteristics of eight ALK-positive cases by IHC

Histopathological diagnosis	Localization of ALK IHC staining	% of positive cells	Staining intensity	FISH	NGS findings
Myoepithelial carcinoma	Membranous	35%	3	Negative	BFAR c.1780G>A NF1 c.7057_7059de
Adenoid cystic carcinoma	Cytoplasmic	15%	1	Negative	ERBB2 c.601G>A HRAS c.484G>A KDR c.1298A>G PTCH1 c.1348_splice RPTOR c.2152C>T TP53 c.712T>A TSC1 c.1060G>A
Basal cell adenocarcinoma	Cytoplasmic	50%	2	Negative	Negative
Adenoid cystic carcinoma	Cytoplasmic Nuclear	70% 5%	3 1	Negative	Not diagnostic
Cribriform adenocarcinoma of minor salivary glands	Nuclear	30%	2.3	Negative	Low-level amplification of ALK and ERBB2
Adenoid cystic carcinoma	Cytoplasmic	15%	2	Negative	Negative
Intraductal carcinoma /low grade cribriform cystadenocarcinoma	Membranous	100%	3	Positive	MYO18A-ALK
Basal cell adenocarcinoma	Cytoplasmic	15%	2	Negative	Negative

In addition, 30 cases (16.5%) showed weak to moderate staining in up to 10% of tumor cells. These cases were not further analyzed.

### Next generation sequencing

**Fig. 2:**
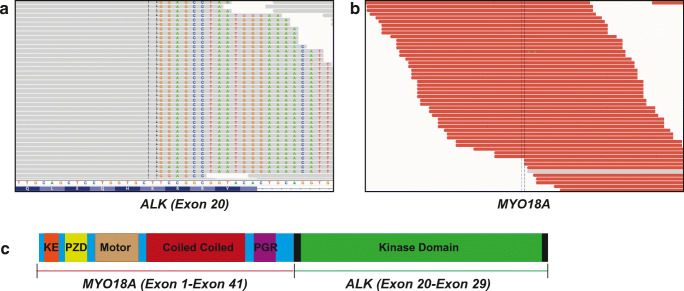
Identification of the novel MYO18A (Exon1-40)-ALK (exon 20-29) gene fusion detected in intraductal carcinoma. **a** Breakpoint spanning reads in exon 20 of ALK. The spanning reads have both aligned (grey) and misaligned bases (colored). The misaligned bases correspond to a region of MYO18A. **b** Encompassing reads in MYO18A (red). The corresponding mate of the reads of MYO18A align to a region in ALK. **c** Schematic representation of the MYO18A-ALK gene fusion

All eight tumors positive for ALK IHC were further analyzed by hybrid capture–based NGS. The FISH ALK break-apart positive tumor was found to harbor a novel *MYO18A*-*ALK* gene fusion (Fig. 2). *The MYO18A-ALK* gene fusion was detected in 71 reads encompassing the breakpoint and 17 reads spanning the breakpoint, thus identifying the exact genomic breakpoint of the fusion. The fusion retains exons 1–41 of *MYO18A* and exons 20–29 of *ALK*, thereby retaining the kinase domain of *ALK* and the coiled-coil domain of *MYO18A*.

Among the other seven ALK IHC-positive cases, there was one additional case that showed a low-level gain in *ALK* with a mean copy number of 4.9 and a low-level *ERBB2* gain with a mean copy number of 5.6. These changes occurred in CAMSG, and this tumor showed medium and strong nuclear IHC ALK positivity in 30% of cells but was negative for *ALK* rearrangement by break-apart FISH analysis.

NGS analysis of the remaining seven ALK IHC-positive samples did not indicate any genomic alterations of *ALK* but resulted in the detection of other important genomic aberrations of potential functional significance, such as inactivating mutations in *BRAF* (p.D594N) and *TP53* (p.C238S), as well as amplifications of *ERBB2* (Table 2).

## Discussion

Genomic *ALK* aberrations are rare in SGCs. Recently, its alterations have been reported in five tumors of this group [18–21]. Out of 182 SGCs we screened by IHC, only eight samples expressed ALK in more than 10% of tumor cells with moderate to strong staining intensity. FISH and hybrid capture–based next generation sequencing analysis performed in these tumors identified one case with a low-level amplification in the *ALK* gene (CAMSG with 30% nuclear positivity). Additionally, one intraductal carcinoma showed *ALK* rearrangement by FISH, in which a hybrid capture–based next generation sequencing identified the exact genomic breakpoint in a novel *ALK* and the *MYO18A* gene fusion on chromosome 17.

Immunohistochemistry (IHC) can be used as a screening method for *ALK* aberrations, although confirmatory genetic testing is recommended [23, 24]. This is also supported by findings in the recent papers [19, 21] in which *ALK-CTTNA1* and *HNRNPH3-ALK* translocations were associated with membranous and cytoplasmic ALK staining, respectively. However, neither IHC nor FISH analysis provides information on the exact breakpoint and on the respective fusion partner. For *ALK*-rearranged tumors, the rearrangement partner may correlate with the intracellular localization of the chimeric protein and the pattern of staining. In tumors with NPM1-ALK fusion, ALK protein may be detected in the nucleus and cytoplasm, while in *RANBP2-ALK* neoplasms, it shows nuclear membrane staining. Fusion proteins TMP3-ALK and CTCL-ALK are found in the cytoplasm, but the former gives diffuse and the latter granular pattern of staining [23]. In our case of IC, the IHC pattern of ALK staining was strongly membranous. In the remaining ALK-positive cases, less intense staining was present in all cellular compartments. This also makes the interpretation of ALK IHC difficult, especially concerning cut-off values that may be taken as an indicator of genetic molecular alterations.

More than 20 different fusion partners of ALK have been described in different neoplasms [23, 25], and their number is still increasing [16, 17, 26, 27]. The type of fusion gene appears to influence not only the subcellular localization but also their biological activity and carcinogenic potential. This variability in localization and activity of *ALK* is highest in anaplastic lymphoma, in which nine fusion variants have been identified so far. However, multiple fusion partners have been also described in inflammatory myofibroblastic tumor, diffuse large B cell lymphoma, and non-small cell lung carcinoma (NSCLC) [25]. These variants have been discovered only recently, and the knowledge on their biological functions is still limited.

*ALK*-rearranged tumors share characteristic clinicopathological features [14, 23, 25, 27]. *ALK*-rearranged lung adenocarcinomas most often affect middle-aged patients and light or non-smokers. Morphologically, some of these tumors display the characteristic cribriform and signet ring morphology with abundant intracellular mucin [28, 29]. Similarly, our case of IC showed a cribriform architecture and focal mucinous differentiation with presence of PAS- and mucicarmine-positive microvacuoles. Whether this feature is associated with *ALK* rearrangement requires studies on a larger series of cases. Taking into consideration the rarity of the tumor, multicenter studies seems to be indicated.

The *MYO18A-ALK* gene fusion consisted of exons 1–41 of *MYO18A* and exons 20–29 of *ALK*, thereby retaining the kinase domain of *ALK* and the coiled-coil domain of *MYO18A*. Based on functional experimental data from similar *ALK* fusions, the coiled-coil domain of MYO18A could potentially mediate the dimerization and activation of MYO18A-ALK, thereby resulting in overexpression of constantly activated protein. However, the biological relevance of the novel *MYO18A-ALK* gene fusion merits further investigation, given potential implications for targeted therapy with ALK inhibitors. In vitro studies might confirm its transforming role, as well as the activation of downstream signaling, and elucidate a possible therapeutic role of different ALK inhibitors. Our patient was not subjected to ALK inhibition as there was no evidence of disease within 8 years after surgery. It is consistent with the indolent clinical behavior characteristics of this tumor as was also reported in another case of ALK-positive IC [20]. However, in the case of SDC with *EML4-ALK* rearrangement, target treatment resulted in acquired ALK G1202R mutation that led to secondary resistance to ALK inhibitor [21].

The screening of seven additional non-invasive ICs did not disclose ALK positivity by IHC. This suggests lack of the *ALK* translocation in these tumors and indicates that the *MYO18A-ALK* gene fusion may be a rare event in these neoplasms. Only analyses of the large series could definitively determine occurrences of this translocation in this tumor type. Interestingly, another translocation in IC was reported by Weinreb et al. [30], who showed *NCOA4-RET* fusion in one case by NGS and *RET* rearrangement in two additional cases by FISH in a series of 19 tumors. Recently, Skalova et al. reported *NCOA4-RET* fusion transcript in 11 cases of intercalated-type IC by NGS [31, 32]. In addition, a novel *TRIM27-RET* fusion transcript was identified in two ICs with apocrine features. Of note, *RET* gene was a part of the NGS gene panel used in our study (supplementary data), but no *RET* changes were detected in the studied cases.

An additional interesting finding in our study was the low-level gains in *ALK* found in the CAMSG. The *PRKD3* translocation was detected in this tumor by FISH [22]. In this multicenter study, *PRKD1*-*3* translocation was found in more than 80% of CAMSGs [33]. In some of those cases, *ARID1A* or *DDX3X* was the translocation partner. CAMSG is also a rare entity, with approximately 70 cases described so far. Low-level *ALK* copy number gain is relatively common in some tumor types, e.g., in alveolar rhabdomyosarcoma [34, 35], in NSCLC [36], and in up to 10% of renal cell carcinomas [37]. They often reflect chromosome 2 polysomy, rather than true focal amplification [23]. The clinical significance and therapeutic relevance of this finding appear limited. Furthermore, a selection of genomic alterations known from other tumor entities was found in the subset of samples analyzed using hybrid capture–based next generation sequencing. Considering the low number of samples tested comprehensively, it is tempting to speculate that SGCs might harbor more targetable alterations (such as *ERBB2* amplifications).

To summarize, we presented a unique extensive analysis of the genetic profile of the salivary gland malignancies showing ALK rearrangements as a rare aberration in these tumors.

## Supplementary Information

ESM 1 (XLSX 23 kb).

ESM 2 (DOCX 13 kb).

## Data Availability

The sequencing data was deposited in the NCBI Sequence Read Archive to be released upon publication (BioProject Accession: PRJNA646228).
