# Supplementary material for: ALK alterations in salivary gland carcinomas
Source: Virchows Arch. 2020 Nov 25;478(5):933–41. doi: 10.1007/s00428-020-02971-w (PMC8099847; doi:10.1007/s00428-020-02971-w)
Supplement: Supplementary file 2 — (DOCX 13 kb). [file 428_2020_2971_MOESM2_ESM.docx]

Supplementary tables: NGS tested genes.

| ABL1 | CD74 | FGFR3 | KIF5B | NRAS | RICTOR |
| --- | --- | --- | --- | --- | --- |
| ALK | CDH1 | FGFR4 | KIT | NRG1 | ROS1 |
| APC | CDK4 | GNA11 | KRAS | NTRK1 | RPTOR |
| AR | CDK6 | GNA13 | MAP2K1 | PDGFRA | SMO |
| ARAF | CDKN2A | GNAI2 | MAP2K2 | PDGFRB | STK11 |
| ATM | CDKN2B | GNAQ | MDM2 | PIK3CA | TP53 |
| ATR | CTNNB1 | GNAS | MET | PIK3R1 | TSC1 |
| BCL6 | DDR2 | GNAT2 | MTOR | PRKDC | TSC2 |
| BRAF | EGFR | HRAS | MYC | PTCH1 | VHL |
| BRCA1 | EML4 | IDH1 | MYCL1 | PTEN |  |
| BRCA2 | ERBB2 | IDH2 | MYCN | RAD50 |  |
| BRD4 | ERBB3 | JAK2 | NF1 | RAF1 |  |
| CCND1 | FGFR1 | KDR | NFE2L2 | RB1 |  |
| CCNE1 | FGFR2 | KEAP1 | NOTCH1 | RET |  |
